# Bibliographical Notices

**Published:** 1853-05

**Authors:** 


### BIBLIOGRAPHICAL NOTICES.

*Letters on Syphilis.* By Ph. Ricord, Chirurgien de l'Hôpital du Midi, &c., &c. With an Introduction by Amédée Latour Translated by W. P. Lattimore, M. D. Philadelphia: A. Hart, 1852. pp. 270.

These Letters appeared originally in the *Union Médicale* of Paris, during the years 1850, 1851. They have found their way, through various channels, to the English reader, and have, since the completion of the series in the Paris Journal, been collected and translated by Dr. Lattimore. They present a resumé of M. Ricord's peculiar views on the subject of syphilis, and are more particularly occupied with the defence and illustration of those "new doctrines" which, he complains, have failed, in the twenty years of his teachings and writings, to penetrate the minds of all his contemporaries. The Letters, however, despite the popularity with which they have been received, will, we think, fail to silence the dissenters from some of M. Ricord's dogmata. He has, indeed, repeated his old views in a very lively and attractive form; but we doubt whether the style and tone of the Letters will add anything to his reputation or give new force to his opinions.

M. Ricord's researches have certainly identified his name with the subject of syphilis. His experiments upon inoculation, his admirable scheme of syphilitic therapeutics, and his application of the speculum to the diagnosis of venereal ulcers, are among the most valuable contributions to modern medicine. But M. Ricord is a man of one idea—and that idea is *inoculation.* He has found it to be a *positive* test of the specific character of the chancrous virus, and he attempts to pervert it into a *negative* test of the non-specific nature of gonorrhœa or, as he denominates it, blennorrhagia. The latter is scarcely less a point with him than the former; and, in fact, more than one-third of the Letters are devoted to it. It is not our purpose to follow M. Ricord's argument on this question. Its weakness strikes us as apparent in the postulate, upon which his facts are brought to bear. "If blenorrhagia," he assumes, "recognizes a specific cause, the muco-pus which it secretes will, undoubtedly, when inoculated, produce phenomena similar to those produced by the inoculation of chancrous pus." (p. 44.) That is, it is enough for M. Ricord to establish that inoculation with the matter of blennorrhagia is not followed by the phenomena which result from inoculation with chancrous pus, to warrant him in inferring the non-specific character of the former secretion!

It has not been our good fortune to read the Letters in the original. The translation is, we dare say, faithful and accurate, but it is evidently the work of an unpractised pen.

---

*Proceedings of the Medical Association of the State of Alabama, at its Sixth Annual Meeting, held December*, 1852. pp. 168.

We have already had occasion to notice the spirit and zeal evinced in the annual Proceedings of the Alabama State Medical Association. The volume just issued maintains the high rank which the profession of Alabama had previously assumed among our State organizations.

